# Comparative In Vivo Analysis of the Integration Behavior and Immune Response of Collagen-Based Dental Barrier Membranes for Guided Bone Regeneration (GBR)

**DOI:** 10.3390/membranes11090712

**Published:** 2021-09-15

**Authors:** Milena Radenković, Said Alkildani, Ignacio Stoewe, James Bielenstein, Bernd Sundag, Olaf Bellmann, Ole Jung, Stevo Najman, Sanja Stojanović, Mike Barbeck

**Affiliations:** 1 Department for Cell and Tissue Engineering, Faculty of Medicine, University of Niš, 18000 Niš, Serbia; milena1390nis@gmail.com (M.R.); stevo.najman@gmail.com (S.N.); s.sanja88@gmail.com (S.S.); 2 BerlinAnalytix GmbH, 12109 Berlin, Germany; said.alkildani@berlinanalytix.com; 3 Clinic and Policlinic for Dermatology and Venereology, University Medical Center Rostock, 18057 Rostock, Germany; ignacio.stoewe@gmx.net (I.S.); james.bielen@gmail.com (J.B.); b.sundag@gfn-selco.de (B.S.); ole.tiberius.jung@gmail.com (O.J.); 4 Research Institute for Farm Animal Biology (FBN), W. Stahl Allee 2, 18196 Dummerstorf, Germany; bellmann@fbn-dummerstorf.de; 5 Department of Biology and Human Genetics, Faculty of Medicine, University of Niš, 18000 Niš, Serbia; 6 Department of Ceramic Materials, Institute for Materials Science and Technologies, Technical University of Berlin, 10623 Berlin, Germany

**Keywords:** collagen-based membranes, Guided Bone Regeneration (GBR), subcutaneous implantation, cross-linking, immune response, porcine-based collagen, barrier membrane, sugar cross-linking

## Abstract

Collagen-based resorbable barrier membranes have been increasingly utilized for Guided Bone Regeneration (GBR), as an alternative to non-resorbable synthetic membranes that require a second surgical intervention for removal. One of the most important characteristics of a resorbable barrier membrane is its mechanical integrity that is required for space maintenance and its tissue integration that plays a crucial role in wound healing and bone augmentation. This study compares a commercially available porcine-derived sugar-crosslinked collagen membrane with two non-crosslinked collagen barrier membranes. The material analysis provides an insight into the influence of manufacturing on the microstructure. In vivo subcutaneous implantation model provides further information on the host tissue reaction of the barrier membranes, as well as their tissue integration patterns that involve cellular infiltration, vascularization, and degradation. The obtained histochemical and immunohistochemical results over three time points (10, 30, and 60 days) showed that the tissue response to the sugar crosslinked collagen membrane involves inflammatory macrophages in a comparable manner to the macrophages observed in the surrounding tissue of the control collagen-based membranes, which were proven as biocompatible. The tissue reactions to the barrier membranes were additionally compared to wounds from a sham operation. Results suggest wound healing properties of all the investigated barrier membranes. However, the sugar-crosslinked membrane lacked in cellular infiltration and transmembraneous vascularization, providing an exclusive barrier function in GBR. Moreover, this membrane maintained a similar swelling ratio over examined timepoints, which suggests a very slow degradation pattern and supports its barrier function. Based on the study results, which showed biocompatibility of the sugar crosslinked membrane and its stability up to 60 days post-implantation, it can be concluded that this membrane may be suitable for application in GBR as a biomaterial with exclusive barrier functionality, similar to non-resorbable options.

## 1. Introduction

The so-called Guided Bone Regeneration (GBR) approach represents procedures in ridge augmentation or bone regeneration [1]. In that context, barrier membranes should act as a barrier in the course of GBR, preventing the collapse of surrounding soft tissue into the bony defects and to ensure the fixation of the implanted bone substitute material (BSM), creating a microenvironment suitable for bone remodeling support [1]. Thereby, this membrane class, as well as other biodegradable polymeric membranes, eliminates the need for a second surgical procedure for biomaterial extraction even compared to non-resorbable materials such as membranes based on polytetrafluoroethylene (PTFE) [2]. Although biodegradable synthetic membranes made of polyesters, polyglycolides, and polylactides were also developed for alveolar ridges preservation, their usage has been associated with disadvantages such as inflammatory reactions or premature resorption leading to negative impact on bone formation [3]. In contrast, collagen-based membranes can also serve in processes of Guided Tissue Regeneration (GTR) as guidance for cell attachment, proliferation, and migration, or to establish transmembraneous nutrient and blood transport, as well as ingrowth of blood vessels leading to “transmembraneous vascularization” [4].

Thereby, it has been described that collagen membranes for GBR should optimally integrate within the host tissue by activating fibroblasts, macrophages, eosinophilic granulocytes, which has supposed to constitute collagen “turn-over” and should result in a replacement of the implanted membranes by vital soft tissue [5]. In contrast, membranes that induce a foreign body reaction, including biomaterial-induced multinucleated giant cells (BMGCs), have been described as “bioincompatible” biomaterials [6]. However, most of the collagen membranes that are used in daily clinical practice induce an inflammatory tissue reaction that leads to their “turn-over” via the cellular elements of the foreign body response [7]. The immune mechanisms underlying the cell-mediated degradation of collagen-based membranes include mainly the activation of phagocytic cells such as macrophages [8]. Activated macrophages in the processes of biomaterial degradation are classified into two phenotypes: pro-inflammatory M1 macrophages (classically activated macrophages) or anti-inflammatory M2 (alternatively activated macrophages), based on their expression profile [9]. The M1 phenotype has shown to be predominantly present in the early phase, typically, at 3–4 days after injury, and participate in material degradation, and then change their polarization to M2 phenotype leading to tissue healing [10,11,12].

Moreover, it has been revealed that the low cell membrane capacity of macrophages leads to their fusion into BMGCs that enable the organism to phagocytose bigger foreign bodies or biomaterials/biomaterial fragments [8,13]. However, their occurrence within an implantation bed of a biomaterial was long considered an indicator of the bioincompatibility of a material [14,15,16]. However, recent results substantiate that these cells express both pro- and anti-inflammatory molecules within the implantation bed of different biomaterials [17,18]. Moreover, it has been shown that the presence of BMGCs can lead to membrane fragmentation and disintegration associated with premature ingrowth of surrounding host tissue, while the BMGC induction was associated with an increased transmembraneous vascularization [10,11,18].

In this context, reaching the balance between the time of cellular material degradation and the tissue healing process guided a resorbable GBR membrane is a serious challenge. Altogether, it is desirable to have membrane resorption in vivo between 4 weeks and a few months due to the clinical goal that needs to be accomplished [19]. Clinical indications such as large volume and multidimensional jawbone defects require membranes with a longer standing time [20,21,22]. The selection of other origin tissue such as dermis, tendons, or the pericardium and also different animal sources, i.e., porcine vs. bovine membranes, has shown to allow to have an influence on the degradation behavior and the tissue reactions with varying degrees of success [11,23,24].

Interestingly, numerous cross-linking techniques were developed to extend and control the durability of collagen-based membranes [7]. Chemical cross-linking with chemical agents such as aldehydes can increase the mechanical strength and life of collagen-based biomaterials but can also lead to partial cytotoxicity [6,25,26].

A further method to prolong the barrier functionality is the cross-linking of collagen with natural sugar–ribose in a process named glycation [23]. This natural reaction of collagen during aging changes cell-collagen interactions, migration, and adhesion patterns and leads to the accumulation of glycation end products (AGEs) [23]. These products have an impact on the resorption activity of osteoclasts, their differentiation pattern, and increasing collagen fibril stiffness, as well as bone fragility [24]. Additionally, the prolonged time of the sugar crosslinked membrane of porcine origin and its ossification was detected in vivo in bone defects of canine and humans [25,26]. However, the type of inflammatory tissue response, as well as a cellular reaction on ribose crosslinked membranes, are still insufficiently described.

Thus, the aim of this study was to examine the integration behavior and immune responses to a porcine-derived sugar crosslinked collagen membrane compared with native collagen membranes of the same animal species. This was achieved by subcutaneous implantation of membranes in rats and subsequent histological and histomorphometrical analysis by already established methods [27].

## 2. Materials and Methods

### 2.1. Membranes

In this study, 3 commercially available collagen-based membranes were tested. The biomaterials were used as they were received without any further modification(s).

#### 2.1.1. Ossix^®^ Plus Membrane

Ossix^®^ Plus (Datum Dental Biotech, Lod, Israel) is a collagen membrane of porcine origin containing collagen type I, obtained from the tendon. This membrane is created from repolymerized collagen monomers, sugar crosslinked in a physiological process involving natural sugar ribose, and sterilized with ethylene oxide. This membrane is designed for GBR indication, resorbed after 8 months in vivo [28], and nonporous.

#### 2.1.2. Bio-Gide^®^ Membrane

The Bio-Gide^®^ membrane (Geistlich Biomaterials, Wolhusen, Switzerland) is composed of collagen type I and III of porcine origin. The biomaterial is obtained from native collagen of the dermis via decellularization, manufactured without cross-linking or chemical treatment post purification, and sterilized by gamma irradiation. This membrane is designed for GBR indications, resorbed after 8 weeks in vivo [29,30], porous, and bilayered.

#### 2.1.3. Jason^®^ Membrane

The Jason^®^ membrane (botiss biomaterials GmbH, Berlin, Germany) is based on native type I and III collagen obtained from decellularized porcine pericardium, is not crosslinked or chemically treated post purification, and sterilized with ethylene oxide. This membrane is designed for GBR indications, completely resorbed after 12 weeks in vivo [31], and porous. 

### 2.2. Histological Characterisation of Membranes

To examine the (ultra-) structure and the thickness of the 3 membranes, a histological preparation of the blank materials was conducted, as previously described [11]. Briefly, the preparation included the histological workup by initial fixation of 3 membrane specimens of each material (sized 10 × 10 mm) in 4% buffered formalin for 24 h followed by dehydration in a series of alcohol and xylene. After paraffin embedding, the blocks were sectioned, resulting in slices with a thickness of 3–5 µm, which were then deparaffinized and histochemically stained via hematoxylin and eosin (H&E). This procedure allowed us to examine and visualize the (ultra-) structures of the biomaterials via a Panthera U light microscope (Motic, Xiamen, China) and a connected Axiocam 105 color camera combined with the software ZEN Core (both: Zeiss, Oberkochen, Germany). Moreover, it allowed the measurement of the membranes’ baseline thicknesses, as described below.

### 2.3. In Vivo Study

#### 2.3.1. Experimental Animals

The preclinical study was performed on 57 Wistar male rats weighing 220–240 g, 10–12 weeks old, which were obtained from the Military Medical Academy (Belgrade, Serbia) and housed at the Faculty of Medicine in the University of Niš (Niš, Serbia), where the study was conducted. The accommodation of animals was carried out under standard conditions of the day-night regime and ad libitum access to food and water.

Prior to the study conduct, it was authorized by the Institutional Ethical Committee of the Faculty of Medicine (University of Niš, Niš, Serbia), based on decision number 323-07-09101/2020-05/5, of the Veterinary Directorate of the Ministry of Agriculture, Forestry and Water Management of the Republic of Serbia (date of approval: 26 August 2020). All procedures in this experiment were managed in accordance with the Animal Welfare Act of the Republic of Serbia. Ordinary pre-and postoperative care was fulfilled following all principles of Animal Health and Welfare.

#### 2.3.2. Study Design

After the implantation procedure, animals were divided into 4 experimental groups, by simple randomization method, as previously described [32], based on the respective biomaterial type and the control group as follows: Ossix^®^ Plus membrane (OP group), Bio-Gide^®^ membrane (BG group), Jason^®^ membrane (JM group) and sham-operated animals (SO group).

Each of the 3 study groups contained 15 animals; 5 animals (*n* = 5) were used per examination period (10, 30, and 60 days), while 4 animals (*n* = 4) were used for the control (SO) group per time point. The sample size was based on a power analysis, including an additional drop-out rate of 5% (effect size 1.3, G*Power) [33]. The implantation procedure was performed as previously published by Barbeck et al. [10,11,23]. Biomaterial implantations were conducted into the subcutaneous tissue of rats under anesthesia with a mixture of ketamine (90 mg/kg) and xylazine (25 mg/kg) administered by intraperitoneal injection. After the skin of the animals was shaved and disinfected with povidone-iodine, an incision down to the subcutaneous tissue within the rostral subscapular region was made. Afterward, a subcutaneous pocket was bluntly built by a scissor followed by biomaterials implantation into the pocket. Thereafter, the wounds were sutured.

At the end of experimental terms, the animals from each group were euthanized by an overdose of the previously described anesthetics. Afterward, the explants, including the remnants of the biomaterials and the surrounding host tissue, were extracted and fixed into 4% formalin solution for 24 h and then further processed through increasing ethanol concentrations, cleared in xylene, and embedded in paraffin.

#### 2.3.3. Histology and Immunohistochemistry

To prepare histological slides, the explants were initially cut into 2 segments of identical dimensions and dehydrated using a series of increasing alcohol concentrations. Afterward, the samples were embedded within paraffin. The paraffin-embedded tissue blocks were sectioned with a thickness of 3–5 µm. Sections were used for histochemical staining, i.e., hematoxylin and eosin (H&E), and for immunohistochemical staining against CD163 and CD11c molecules. CD163 marker is specific for the anti-inflammatory M2 phenotype of macrophages. CD11c marker is specific for the pro-inflammatory M1 phenotype of macrophages. 

Furthermore, 4 additional sections of every tissue explant were used for the immunohistochemical detection of macrophages and their M1- and M2-subforms by means of antibodies against the pro- and anti-inflammatory molecules, i.e., CD163 and CD11c. Briefly, the slides were initially treated with citrate buffer and proteinase K at pH 8 for 20 min in a water bath at 96 °C, followed by equilibration using TBS-T buffer. Subsequently, the slides were prepared by H_2_O_2_ and avidin and biotin blocking solutions (Avidin/Biotin Blocking Kit, Vector Laboratories, Burlingame, CA, USA). Incubation with the respective first antibody for 30 min was conducted, followed by incubation with the secondary antibody (goat anti-rabbit IgG-B, sc-2040, 1:200, Santa Cruz Biotechnology, Shandon, CA, USA). Afterward, the avidin–biotin–peroxidase complex (ThermoFisher Scientific, Dreeich, Germany) (30 min) was applied, and counterstaining by hematoxylin and blueing was conducted. A detailed process was described by Lindner et al. [27].

#### 2.3.4. Histopathological Analysis

To analyze the samples histologically, the prepared slides were studied using a Panthera U microscope (Motic, Xiamen, China). The analyses were carried out according to the DIN ISO 10993-6 protocol, which was previously described [27]. Briefly, the analyses evaluate tissue-biomaterial interactions within the framework of early and latened tissue response, including parameters such as fibrosis, necrosis, hemorrhage, vascularization; as well as the presence of immune cells (e.g., granulocytes, lymphocytes, macrophages, biomaterial-associated multinucleated giant cells (BMGCs)). Photographs of these interactions were taken using an Axiocam 105 color camera that was connected to its software ZEN Core (Zeiss, Oberkochen, Germany).

#### 2.3.5. Histomorphometrical Analysis

To quantify the occurrence of M1 and M2 macrophages within the implant beds, a digital method using the Image J software (National Institutes of Health, Bethesda, MD, USA) was carried out as previously described [27]. Briefly, the immunohistochemically stained slides were digitalized and inserted to Image J. After manually marking the region of interests (e.g., defect area, membrane, etc.) of 5 samples per time point and group, the number of stained cells (positives to the respective marker) were automatically counted using a specialized plugin as described by Lindner and colleagues [27]. The number of positive cells was related to their respective total areas, and quantification of the number of cells per mm^2^ was obtained.

The thickness of the membranes was measured as follows. The digitalized slides were inserted into Image J and calibrated using the respective scale bar. For each slide, the membrane thickness was measured 5 times at different spots, using straight lines that were perpendicular to the membrane.

#### 2.3.6. Statistical Analysis

The quantitative data were statistically analyzed via an analysis of variance (ANOVA), using the GraphPad Prism 8.0 software (GraphPad Software Inc., La Jolla, CA, USA). Statistical differences were recognized as significant according to the respective *p*-values. The difference was significant if *p*-values were less than 0.05 (* *p* ≤ 0.05), and highly significant if *p*-values were less than 0.01 (** *p* ≤ 0.01), less than 0.001 (*** *p* ≤ 0.001) or less than 0.0001 (**** *p* ≤ 0.0001).

## 3. Results

### 3.1. Histopathological Analysis

**Figure 1:**
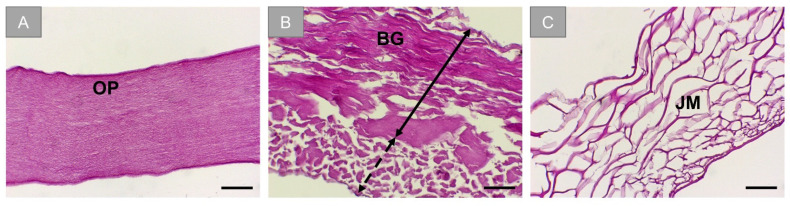
Histological visualization of the barrier membranes. (**A**) Ossix^®^ Plus (OP), (**B**) Bio-Gide^®^ (BG). Black double-headed arrow: compact layer, dotted double-headed arrow: porous layer. (**C**) Jason^®^ membrane (JM). (H&E stainings, scale bars: 50 µm, objective magnification: 20×).

Histological analysis of the membranes showed the initial structural state of the barrier membranes. OP appears to have a nonporous microstructure (Figure 1A). BG exhibits distinctively two different layers, one that is compact and the other porous (Figure 1B). JM exhibits honeycomb porosity (Figure 1C).

**Figure 2:**
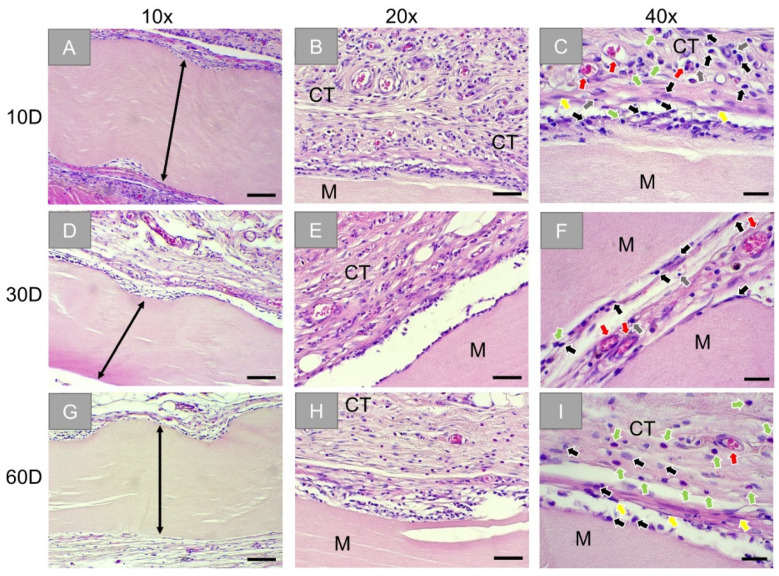
Exemplary histological images of the subcutaneously implanted collagen-based sugar-crosslinked barrier membrane (Ossix^®^ Plus) at three time points: 10 days (first row: (**A**–**C**)), 30 days (second row: (**D**–**F**)), and 60 days (third row: (**G**–**I**)). Stretched black arrow: compact layer of the membrane and M: membrane, CT: connective tissue, black arrows: macrophages, red arrows: blood vessels, green arrows: granulocytes, grey arrows: lymphocytes, yellow arrows: fibroblasts. (HE-stainings, 10×, 20×, and 40× objective magnifications with scale bars: 100 µm, 50 µm, and 20 µm, respectively).

Histopathological analysis of the subcutaneously implanted OP membranes showed that the membranes were found at day 10 (Figure 2A). A slight inflammatory tissue reaction and minimal fibrosis were observed surrounding the implanted biomaterial (Figure 2B,C). This reactive tissue at the surface of the implants constitutes mainly blood vessels and cells of the immune system (i.e., macrophages, granulocytes, and lymphocytes) (Figure 2C). On day 30, the membranes remained intact (Figure 2D). Similar to the previous study time point, the biomaterial was surrounded by blood vessels and reactive tissue that constitutes the same cell types of the immune system (Figure 2F). At day 60 post-implantation, the membranes were still found intact and surrounded by slight fibrosis, blood vessels, and reactive tissue showing a trend to a decreased inflammatory cell reaction (Figure 2G). The reactive tissue remained on the surface of the implants, and an increase in the occurrence of granulocytes surrounding the implant was noticed (Figure 2I).

At all time points, no signs of necrosis were seen within the implantation beds of the OP membranes, and the reactive tissue did not infiltrate the membrane.

**Figure 3:**
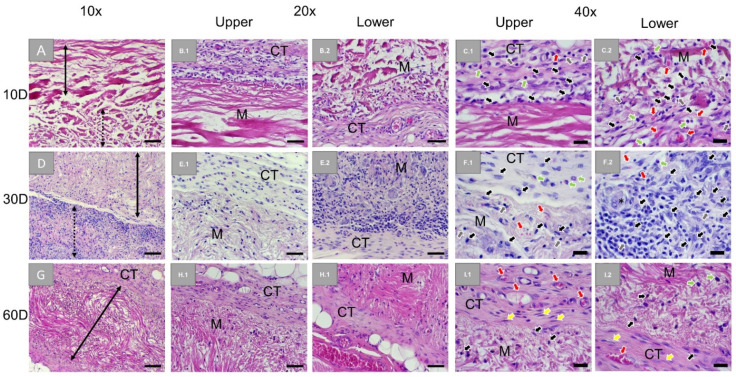
Exemplary histological images of subcutaneously implanted collagen-based bilayer barrier membrane (Bio-Gide^®^) at three timepoints: 10 days (first row: **A**,**B.1**,**B.2**,**C.1**,**C.2**), 30 days (second row: **D**,**E.1**,**E.2**,**F.1**,**F.2**), and 60 days (third row: **G**,**H.1**,**H.2**,**I.1**,**I.2**). Stretched black arrow: compact layer of the membrane, stretched dotted arrow: loose layer of the membrane, CT: connective tissue, M: membrane, black arrows: macrophages, red arrows: blood vessels, green arrows: granulocytes, grey arrows: lymphocytes, yellow arrows: fibroblasts (HE-stainings, 10×, 20×, and 40× objective magnifications with scale bars: 100 µm, 50 µm, and 20 µm, respectively).

Histopathological analysis of the subcutaneously implanted BG membranes showed the intact membrane composed of two distinct layers at day 10 (Figure 3A). A slight inflammatory tissue reaction was seen surrounding the compact layer of the membrane. This reactive tissue was constituted of blood vessels and cells of the immune system (i.e., macrophages, granulocytes, and lymphocytes) (Figure 3B.1,C.1). A similar but slightly pronounced inflammatory tissue reaction was also seen at the loose layer of the membrane with noticeably increasing vascularization (Figure 3B.2,C.2). At day 30, the two layers of the membrane remained distinguishable based on the different rates of cellular infiltration (Figure 3D). Blood vessels and a similar reactive tissue compared to day 10 were seen infiltrating the compact layer (Figure 3F.1). However, the reactive tissue infiltrated the loose layer in an increasing manner, where also biomaterial-associated multinucleated giant cells (BMNCs) were observable (Figure 3F.2). At day 60 post-implantation, the membrane was still intact and infiltrated by blood vessels and a decreased reactive tissue, while slight fibrosis was observable at the material surfaces (Figure 3G–I). The loose membrane layer was no longer detectable, suggesting that it was completely resorbed.

At all timepoints, no signs of necrosis were seen within the implantation beds and the reactive tissue does infiltrate the membrane but with different infiltration degrees depending on the structure of the two layers were observed.

**Figure 4:**
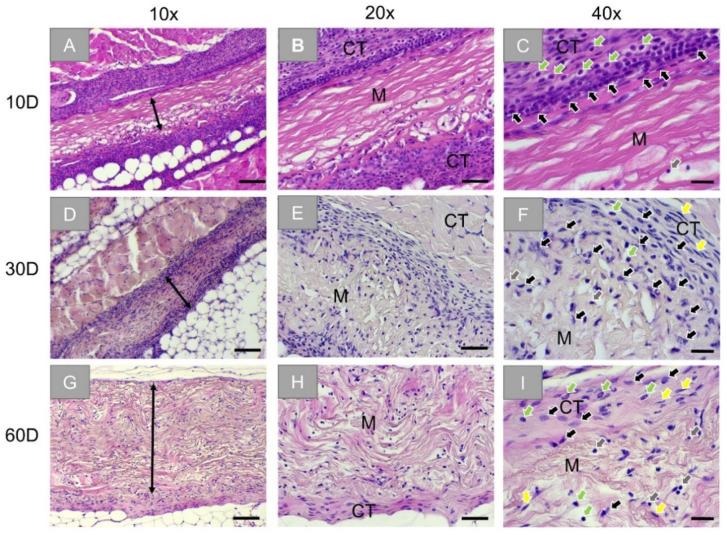
Exemplary histological images of subcutaneously implanted collagen-based non-crosslinked membranes (Jason^®^ membrane) at three timepoints: 10 days (first row: **A**–**C**), 30 days (second row: **D**–**F**), and 60 days (third row: **G**–**I**). Stretched black arrows and M: membrane, CT: connective tissue, black arrows: macrophages, green arrows: granulocytes, grey arrows: lymphocytes, yellow arrows: fibroblasts (HE-stainings, 10×, 20×, and 40× objective magnifications with scale bars: 100 µm, 50 µm, and 20 µm, respectively).

Histopathological analysis of the subcutaneously implanted JM membrane also showed intact membranes at day 10 (Figure 4A). A moderate inflammatory tissue reaction was seen surrounding the implanted biomaterial. This reactive tissue, located on the surfaces of the implants and slightly infiltrating it, was composed of cells of the immune system (i.e., macrophages, granulocytes, and lymphocytes) (Figure 4C). At day 30, the membrane remained intact (Figure 4D). However, the reactive tissue significantly decreased compared to day 10, and slight fibrosis was seen located on the surfaces of the implants (Figure 4E). The reactive tissue seems to infiltrate two-thirds of the membrane from both sides (Figure 4F). At day 60 post-implantation, the membrane was still intact, surrounded by slight fibrosis, and completely infiltrated with the aforementioned reactive tissue (Figure 4G–I).

At all time points, no signs of necrosis were seen within the implantation beds, and the reactive tissue gradually infiltrated the membrane.

**Figure 5:**
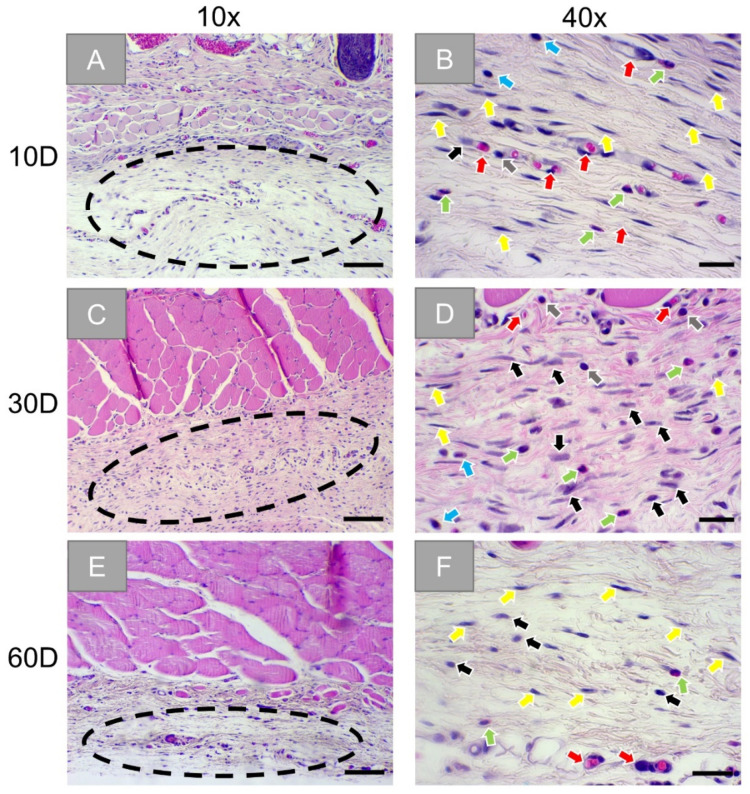
Exemplary histological images of the tissue reactions in the sham operation group at three timepoints: 10 days (first row: **A**,**B**), 30 days (second row: **C**,**D**), and 60 days (third row: **E**,**F**). Dotted circles: wound areas, black arrows: macrophages, red arrows: blood vessels, green arrows: granulocytes, grey arrows: lymphocytes, yellow arrows: fibroblasts, blue arrow: plasma cells. (HE-stainings, 10× and 40× objective magnifications with scale bars: 100 µm and 20 µm, respectively).

Finally, the histopathological analysis of the slides from the sham operation group showed a mild inflammation tissue reaction within the wound areas (Figure 5A). In concert with blood vessels, mainly macrophages, granulocytes, plasma cells, lymphocytes, and fibroblasts were observed (Figure 5B). At day 30, a very similar tissue reaction was still seen (Figure 5C,D). The wound site seems to be healed at day 60 with only slight signs of inflammatory reactive tissue present (Figure 5E,F).

#### Immune Response

**Figure 6:**
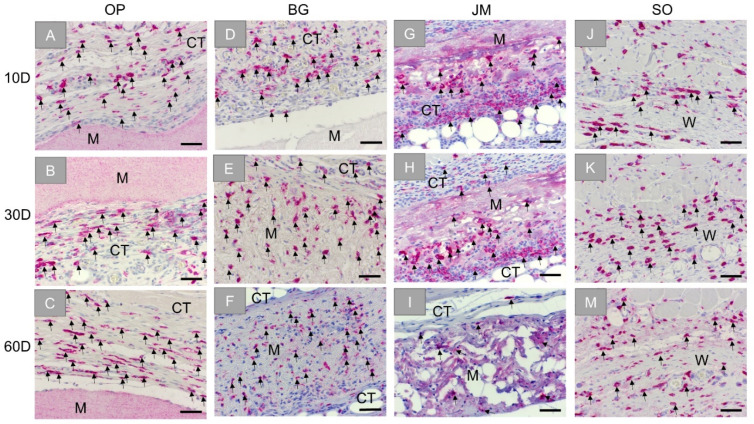
Exemplary images of the immunohistochemical detection of anti-inflammatory M2 macrophages within the bed implants of the different barrier membranes and the sham operation group at three time points: 10 days, 30 days, and 60 days. First column: Ossix^®^ Plus (OP) (**A**–**C**), second column: Bio-Gide^®^ (BG) (**D**–**F**), third column: Jason^®^ membrane (JM) (**G**–**I**), and forth column: sham operation (SO) (**J**–**M**). CT: connective tissue, M: membrane, W: wound area, black arrows: CD163-positive cells (CD163-immunostainings, 20× objective magnifications and scalebars = 50 µm).

The analysis of the immunohistochemical detection of anti-inflammatory CD163-positive macrophages showed high numbers of M2 macrophages within the reactive inflammatory tissue that surrounded the Ossix^®^ Plus membrane at day 10 (Figure 6A). Comparable numbers of M2 macrophages were also observable within the reactive tissue at the later timepoints, located on the surface of the membrane and within the surrounding reactive tissue (Figure 6B,C).

In addition, in the group of the Bio-Gide^®^ membrane, CD163-positive M2 macrophages were found mainly located within the reactive tissue surrounding the implant at day 10 (Figure 6D). In an increasing fashion, the CD163-positive cells were found within the adherent connective tissue, and the M2-macrophages were infiltrating the membrane at the following study time points (Figure 6E,F).

In case of the Jason^®^ membrane, relatively high numbers of M2 macrophages were also seen, especially at the material surfaces but also within the reactive tissue surrounding the implant at day 10 (Figure 6G). In an increasing fashion, the CD163-positive cells were seen infiltrating the membrane at the advanced timepoints (Figure 6H,I).

Finally, moderate numbers of CD163-positive M2 macrophages were detected within the wound area of the sham operation, maintaining comparable numbers for all time points (Figure 6J–M).

**Figure 7:**
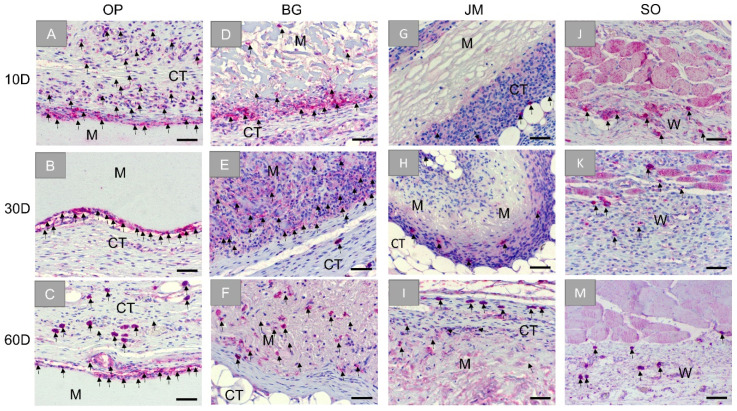
Exemplary images of the immunohistochemical detection of pro-inflammatory M1 macrophage within the bed implants of the different barrier membranes and the sham operation group at three time points: 10 days, 30 days, and 60 days. First column: Ossix^®^ Plus (OP) (**A**–**C**), second column: Bio-Gide^®^ (BG) (**D**–**F**), third column: Jason^®^ membrane (JM) (**G**–**I**), and forth column: sham operation (SO) (**J**–**M**). CT: connective tissue, M: membranes, W: wound area, black arrows: CD11c-positive cells (CD11c-immunostainings, 20× objective magnifications and scalebars = 50 µm).

Additionally, the histopathological analysis based on the immunohistochemical detection of pro-inflammatory CD11c-positive macrophages showed that this cell type was mainly located at the material surfaces and within the reactive inflammatory tissue that surrounds the Ossix^®^ Plus membrane at day 10 (Figure 7A). Similar to that, the M1 macrophages were seen at the membrane surfaces and within the reactive tissue at the later time points (Figure 7B,C).

In the group of the Bio-Gide^®^ membrane, M1 macrophages were seen within the reactive tissue surrounding the implant but especially at the membrane surfaces at day 10 (Figure 7D). Prominently, these cells were located at the surface of the loose layer of the membrane. M1 macrophages are also detected within the membranes and the surrounding connective tissue at later time points (Figure 7E,F).

In the case of the Jason^®^ membrane, CD11c-positive M1 macrophages were scarcely seen within the surrounding reactive tissue at day 10, while low numbers of this cell type were additionally found infiltrating the membrane at later study time points (Figure 7G–I).

Finally, CD11c-positive M1 macrophages were infrequently found within the wound area of the sham operation, maintaining incomparable occurrences to M2 cells for all time points (Figure 7J–M).

### 3.2. Histomorphometrical Results

**Figure 8:**
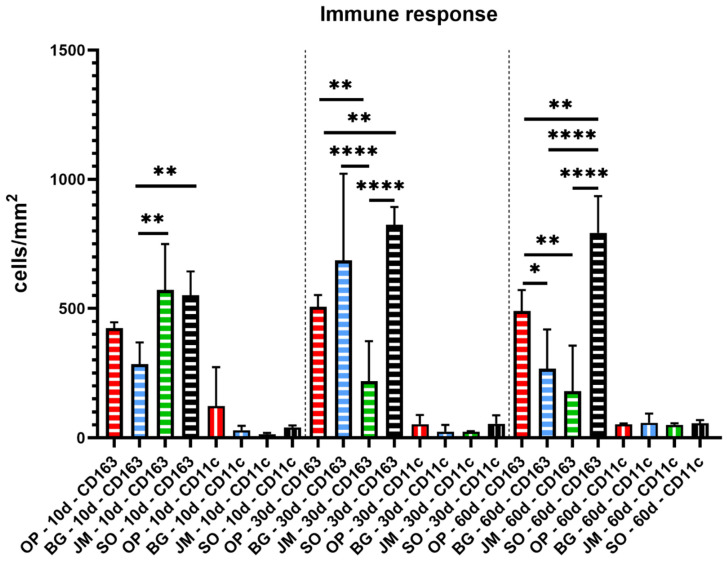
Statistical analysis of the histomorphometrical results of the immune response within the implantation bed of the analyzed barrier membranes (Ossix^®^ Plus (red), Bio-Gide^®^ (blue), Jason^®^ membrane (green)) and of the sham operation (black). Vertical stripes: CD11c and horizontal stripes: CD163. (* *p* ≤ 0.05, ** *p* ≤ 0.01, **** *p* ≤ 0.0001).

The results of the histomorphometrical analysis are presented in Table 1 and Figure 8 that display only the interindividual significant differences. Additionally, intraindividual significances between the M1 and M2 cell counts at the different study time points within the same study group are shown in Table 2.

On day 10, comparable numbers of M1 macrophages without significant differences were found in all study groups. Furthermore, comparably high numbers of M2 macrophages were found in the groups of the Jason membrane and the sham operation that were significantly higher compared to the numbers in the Bio-Gide^®^ group (** *p* ≤ 0.01) (Table 1 and Figure 8). However, no statistical differences were found compared to the numbers in the Ossix^®^ Plus group at this early time point. Furthermore, no differences were found comparing the values in the Bio-Gide^®^ group and the Ossix^®^ Plus group (Table 1 and Figure 8). Moreover, the numbers of M2 macrophages were significantly higher in all study groups compared to the numbers of M1 macrophages at day 10 (Table 2).

On day 30, still no significant differences between the numbers of M1 macrophages were found comparing all study groups (Table 1 and Figure 8). At this time point, the numbers of M2 macrophages were found comparable in the groups of the Bio-Gide^®^ membrane and the sham operation group, which were both significantly higher compared the numbers in the Jason^®^ membrane group (**** *p* ≤ 0.0001). Furthermore, the numbers of M2 macrophages in the sham operation group were significantly higher compared to the values in the Ossix^®^ Plus membrane group (** *p* ≤ 0.01), whose numbers were also higher compared to that in the Jason^®^ membrane group (** *p* ≤ 0.01) (Table 1 and Figure 8). Moreover, the numbers of M2 macrophages were significantly higher in all study groups compared to the numbers of M1 macrophages at day 30 (Table 2).

On day 60, still no significant differences between the numbers of M1 macrophages were found comparing all study groups (Table 1 and Figure 8). The numbers of M2 macrophages in the sham operation group were significantly higher (** *p* ≤ 0.01 and **** *p* ≤ 0.0001) compared to all three membrane groups at this time point (Table 1 and Figure 8). Furthermore, the M2 macrophage numbers in the Ossix^®^ Plus group were significantly higher (* *p* ≤ 0.05 and ** *p* ≤ 0.01) compared to the values in the groups of the Bio-Gide^®^ membrane and the Jason^®^ membrane (Table 1 and Figure 8). At this time point, the numbers of M2 macrophages were significantly higher in all study groups compared to the numbers of M1 macrophages with the exception of the Jason^®^ membrane group that induced comparable numbers of both cell types at this time point (Table 2).

**Figure 9:**
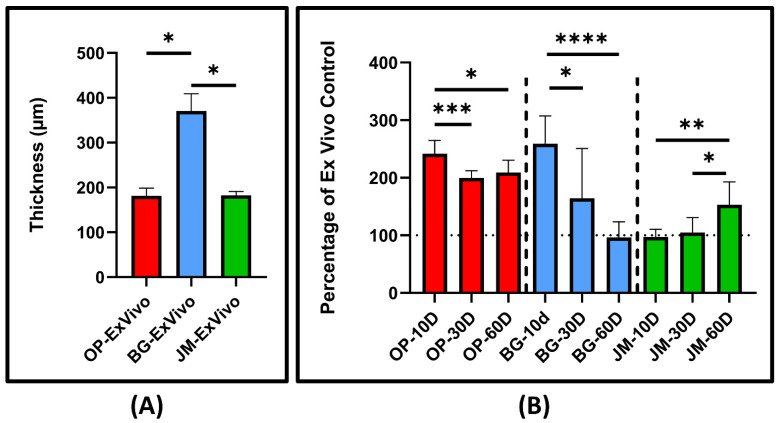
Statistical analysis of the histomorphometrical results of the ex vivo (**A**) and in vivo (**B**) thickness of the barrier membranes (Ossix^®^ Plus (red), Bio-Gide^®^(blue), and Jason^®^ membrane (green)) at different time points. The measurements are relative percentages of the ex vivo baseline membranes. (* *p* ≤ 0.05, ** *p* ≤ 0.01, *** *p* ≤ 0.001, **** *p* ≤ 0.0001).

As for the membrane thickness, the histomorphometrical analysis is displayed in Figure 9A, describing the ex vivo baseline thickness of each investigated membrane. Figure 9B displays the percentage thickness of the implanted membranes relative to the initial ex vivo thickness.

The initial thickness of Bio-Gide^®^ was significantly larger than both Ossix^®^ Plus and Jason^®^ (* *p* ≤ 0.05). Compared to their baseline thicknesses, OP increased 241% in thickness, BG increased 254%, and JM remained the same at day 10. At day 30, the thickness of OP was 200% larger than its initial measurement, BG 166%, and JM remained the same. For the last time point, after 60 days, OP measured 209% of its initial thickness, BG 97%, and JM 152%. Control percentages of OP measured significant differences between day 10/day 30 and day 10/day 60 (*** *p* ≤ 0.001 and * *p* ≤ 0.05, respectively). Control percentages of BG measured significance differences between day 10/day 30 and day 10/day 60 (* *p* ≤ 0.05 and **** *p* ≤ 0.0001, respectively). Finally, control percentages of JM measured significance differences between day 10/day 60 and day 30/day 60 (** *p* ≤ 0.01 and * *p* ≤ 0.05, respectively).

## 4. Discussion

Various collagen-based membranes are available for GBR procedures in the fields of oral, and maxillofacial surgery [34]. Although resorbable collagen-based membranes are favored due to the avoidance of a second surgery, some clinical applications require space maintenance of the barrier membrane with stronger tensile strength or a prolonged biodegradation time [35,36,37]. The prolonged barrier functionality is usually achieved by the application of non-resorbable synthetic materials based on titanium or PTFE [38,39]. On the other hand, resorbable collagen crosslinked membranes are developing, with production techniques that may lead to an extended-standing time in concert with acceptable inflammatory tissue reactions [37,38,39]. Since non-degradable synthetic materials require removal, which leads to repetitive tissue trauma, and many crosslinked techniques have been shown to be cytotoxic or bioincompatible, there is a need for a production technique leading to the desired integration pattern combined with an adapted degradation pattern and a desired inflammatory tissue response [6,25,26]. Since glycation is a natural reaction of collagen fibers during aging, it is supposed to be an optimal basis for sugar crosslinking of collagen, resulting in a barrier membrane that may provide a sufficiently long barrier functionality and manifest integrative behavior, which is not harmful to the peri-implant tissue [24]. Thus, the present study was conducted to examine the integration behavior, and immune response of a sugar crosslinked membrane of porcine origin by established histopathological and histomorphometrical analysis methods for immunohistochemical detection of macrophages subtypes M1 and M2. The two barrier membranes that are based on native collagen of porcine origin and that are proven as a biocompatible and resorbable were used as control biomaterials. A concluding table below provides a comparison of the similarities and differences between the investigated barrier membrane, Ossix^®^ Plus, and the controls (Table 3).

Initially, the histological analysis of the blank membranes showed different microstructures. Ossix^®^ Plus is seen to be nonporous, which provides an exclusive barrier function. Bio-Gide^®^ showed two distinctive layers, one that is compact, and the other is loose and porous. The manufacturer of Bio-Gide^®^ recommends the application of the porous layer towards the bone to allow enhanced cellular integration, providing an osteoconductive micromilieu for bone growth [30]. Jason^®^ membrane exhibited a honeycomb-like porosity, which allows for cellular infiltration. Ultimately, the understanding of the original condition of each barrier membrane provides insight into their integration patterns that include cellular infiltration, vascularization, and degradation, which are all essential properties of barrier membranes for GBR, and these variabilities might also influence clinical outcomes in terms of wound healing and bone augmentation. Another distinguishing factor of resorbable barrier membranes compared to non-resorbable PTFE, is having not only a barrier function but also soft tissue integration that can potentially enhance the aforementioned clinical outcomes [4].

The histological analysis of the integration pattern of the three membranes revealed that the integration pattern of the Ossix^®^ Plus membrane is completely different from both native collagen membranes since it does not undergo integration with the surrounding tissue. In this context, the main functionality of every GBR membrane is space maintenance, which can be achieved in the case of all analyzed membranes as the three materials remained stable without any signs of membrane fragmentation up to the end of the observation period. However, for some time now, further demands have been made on collagen-based GBR membranes, including transmembraneous vascularization [40]. The results of the present study show that the sugar crosslinked membrane does not allow for this material parameter, which might decrease their regenerative potential in comparison to the conventional collagen-based membranes included as control materials in this study. However, this integration pattern is comparable with non-resorbable barrier membranes but with the advantage of lowering the risk of soft tissue dehiscence [38], which has been frequently reported with PTFE-based barrier membranes. Bio-Gide has also been shown to lower the risk of dehiscence [41].

Finally, the question about the degradation time of this membrane type arises as this factor is also of high importance for the bone regeneration process [42]. Another related issue of this membrane might be seen in the prolonged standing time combined with the observed degradation resistance, as this material characteristic might lead to “intrinsic” pressure onto the overlying mucosal flap, which means that even in cases with a reduced flap tissue, the application of this membrane might be challenging. This assumption is furthermore underlined by the material analyses that have been shown that the Ossix^®^ Plus membrane excelled with the increased fragility in dry but also wet conditions, probably due to the applied crosslinked technique by sugar, in comparison with the native collagen-based membrane Bio-Gide^®^, which had more elasticity [43]. These observations were additionally supported by the histomorphometrical analysis of the material thickness that showed an approximate swelling of the crosslinked membrane up to 250% after 10 days post-implantation that was significantly reduced over the study period but remained still at around 200% swelling up to day 60 post-implantation. The swelling in the case of the crosslinked membrane cannot have been caused by cellular infiltration, which leads to the conclusion that this swelling behavior must be mediated by the ingredients. In this context, it is known that the less the collagen is crosslinked, the more this molecule can bind water [44]. Thereby, it was also revealed that collagen hydration varies according to the degree of crosslinking. However, in the case of the ribose-based crosslinking, it is strongly suggestable that this sugar crosslinker is the reason for the increasing water-binding capacity of the membrane and, thus, for its swelling behavior. This behavior was also observed with other sugar-based crosslinkers [45].

In addition, the Bio-Gide^®^ membrane showed a swelling of approximately 260% at day 10 post-implantation that was gradually decreasing over time down to 97% at day 60 post-implantation, indicating a “real” degradation profile. This thickness profile might be induced by the cellular migration, especially in the porous material component that is also described in more detail in the next paragraph, thus that the dense material part is less responsible for this effect. Thus, it is thinkable that the observed decrease in the membrane thickness is correlated with the stepwise degradation and integration of the porous membrane component. In contrast, the Jason^®^ membrane did not show any swelling at day 10 post-implantation compared to its material thickness, but its value significantly increased at day 60 post-implantation. This phenomenon is explainable by the ongoing cellular ingrowth into the body of the membrane, which takes place mainly at later time points.

The histopathological analysis revealed that the Ossix^®^ Plus membrane induced only a slight inflammatory tissue reaction and minimal fibrosis only involving mononuclear cells indicating its good biocompatibility. Thereby, no cell infiltration was observed over the complete study period. In contrast, the tissue response towards the Bio-Gide^®^ membrane was bifurcated as both its disparate layers induced different integration behaviors during the whole experimental period. The peri-implant tissue composed of blood vessels and macrophages, granulocytes, and lymphocytes beside very low numbers of BMGCs infiltrated both layers, but with different time dynamics during the observation time points. The infiltration of the porous layer was observable to a higher extent up to 30 days post-implantation. Furthermore, the porous layer was completely resorbed until 60 days post-implantation, while the compact layer was visible in this time period but infiltrated with cells in a higher range, mostly at this late study time point. Finally, the analysis showed that the Jason^®^ membrane was not resorbed until 60 days post-implantation. The tissue reaction was comparable to that found in the Bio-Gide^®^ membrane group. While mostly mononuclear cells, i.e., mainly macrophages, were found attached to the membrane, also BMGCs were noted in a smaller number within implantation bad of Jason^®^ membrane. However, both membranes based on native collagen showed no sign of material fragmentation or breakdown.

Especially the results in the sugar-crosslinked Ossix^®^ Plus membrane group are interesting as it has been described that chemically crosslinked barrier membranes trigger a foreign body reaction that seems to lead to a premature material fragmentation, which impairs their barrier functionality in terms of GBR [7]. The results of previous examinations revealed that the Ossix^®^ Plus membrane showed prolonged biodegradation and low tissue integration but no foreign body reaction [46,47]. A further explanation for the basis of the observed tissue response without the occurrence of BMGCs might be findable in the manufacturing process of the ribose crosslinked membrane, which is produced via a preliminary stage that includes comminution of the collagen or a collagen solution even in contrast to the other two membranes, which are directly extracted from the donor tissue. In this context, it has been shown in the case of synthetic bone substitute materials that also did not induce BMGCs in contrast to other materials with the same chemical composition that the macrophages were “able to detach individual smaller subunits”, which seem to be phagocytizable by mononuclear cells due to a sufficient membrane capacity [48].

Additionally, the results of our study revealed a high number of granulocytes at the last time point in the Ossix^®^ Plus group. Their occurrence can also be seen as a further indicator of the prolonged and slow-released biodegradation, as it has been shown even in a study analyzing the tissue responses to the membrane Bio-Gide^®^ membrane that this cell type is involved in the physiological transition of collagen-based biomaterials [49,50]. Thus, it is thinkable that the crosslinking of this membrane hinders the release of collagen fragments from this membrane, causing new recruitment of granulocytes to the site at later time points post-implantation. This is supported by the fact that granulocytes are short-lived immune cells [51]. This observation suggests that the biodegradation of this membrane might start at day 60. Additionally, since it has been shown that many pathological conditions in chronic diseases are connected with AGEs accumulation, which naturally occurs during crosslinking of collagen, examination of a possible presence and connection of AGEs on immunological tissue response on a molecular level should be examined [23].

Altogether, it can be concluded that, despite the small differences observed, the main observation of the long-term stability without any cell or tissue ingrowth is comparable to previous in vivo results. Furthermore, the observed integration behavior is in line with different clinical observations that showed successful jawbone regeneration after the application of the Ossix^®^ Plus membrane [25,26]. Although the results of this study showed no real tissue integration of the Ossix^®^ Plus membrane, it has clinically been described that this membrane also undergoes bony integration. These results have to be examined in more detail in new in vivo studies as this tissue integration is contrary to previous assumptions, which included the theory that more bioactive membranes that support, for example, processes such as a transmembraneous vascularization, have to be used for support of the regeneration process [4,25]. This assumption is interesting insofar as this integration behavior is quite different from that of both control materials in the present study that showed a complete integration within the tissue, including the promotion of a transmembraneous vascularization. Interestingly, new study results also show an optimal bony integration of the Jason^®^ membrane (manuscript submitted), which was related to its integration behavior. However, the results of the present study are likely to put these results into perspective.

Another aspect of the present study is the integration behavior of the Bio-Gide^®^ membrane. Interestingly, the difference in cellular infiltration characteristics between two different layers of Bio-Gide^®^ was not described in subcutaneous implantation in Lewis rats, which may be a consequence of implantation on a different animal strain, but also due to the absence of the identical experimental conditions [52]. In contrast, the same integration behavior has already been described for the Jason^®^ membrane in other preclinical studies [40].

Altogether, the question arises if a membrane that induces inflammatory tissue responses and a following higher vascularization or a membrane with an associated slight tissue response are more suitable for the desired clinical result. This topic is also discussed by other different groups, but no final conclusion could be derived until now [4,38].

Finally, the histomorphometrical analysis of the immune response showed that all three membrane types induced a significantly higher occurrence of anti-inflammatory macrophages compared to the pro-inflammatory subtype with an exception in the group of the Jason^®^ membrane at day 60 post-implantation with comparable numbers of both variants. Moreover, no significant differences in the numbers of pro-inflammatory macrophages have been measured between all study groups, i.e., the membrane groups and the sham operation group, at any of the study time points. In contrast, significant differences have been found between numbers of anti-inflammatory macrophage subtypes throughout the whole observation period. Interestingly, the highest M1/M2 differences were found in the sham operation group over the complete study period. This could be explained by the increase of the M1 phenotype in the presence of the implants, supporting the balancing M1/M2 polarization within and around biomaterials that is explained previously [12,16].

In the Ossix^®^ Plus membrane group, consistently similar M2 macrophage values were found over the whole study period that was comparable to that found in the Bio-Gide^®^ membrane group and the Jason^®^ membrane group up to day 30 post-implantation. At day 60 post-implantation, the M1 macrophage numbers in this group were significantly higher compared to the values in the two control groups. These values lead to the conclusion that the Ossix^®^ Plus membrane can induce a microenvironment, including an inflammatory response-oriented to a material-mediated tissue healing.

In addition, the Bio-Gide^®^ membrane induced an intermediate M2 macrophage level with an exception at day 30 post-implantation being comparably high as the values in the sham operation group, but its M2 values were also significantly lower compared to that found in the Ossix^®^ Plus membrane group at day 60 post-implantation. Finally, the values in the Jason^®^ membrane group showed an initial high M2 macrophage occurrence that continuously decreased towards the end of the observation period. Interestingly, the M2 macrophage numbers were significantly higher in the Jason^®^ membrane at day 10 post-implantation, while the values were higher in the Bio-Gide^®^ membrane group at day 30 post-implantation. However, at day 60 post-implantation, the values in both groups were comparable. Despite these minor differences, both membranes also create a comparable regeneration-promoting environment leading to significantly higher anti-inflammatory conditions as expected from an optimal biomaterial. A broad variety of (pre-) clinical studies showed their successful application in terms of GBR, which supports this assumption. Further preclinical in vivo studies have to show the exact differences of the three membranes even in the context of bone healing, but the present results lead to the overall conclusion that all three medical devices are optimally suitable for bone tissue regeneration and even as barrier membranes.

## 5. Conclusions

The examined membranes originate from the same animal species, but they are obtained from different tissues and underwent different manufacturing processes. As a result, data from the present study indicate differences in the integration behavior of sugar crosslinked collagen membrane in comparison with native collagen membranes suggesting that sugar crosslinked collagen membrane should be used as a barrier membrane only. In addition, the immune response to all examined membranes is comparable, indicating that all examined collagen membranes are biocompatible and can be used for Guided Bone Regeneration (GBR) applications.

## Figures and Tables

**Table 1:** Histomorphometrical results of the immune response within the implantation bed of the analyzed barrier membranes (Ossix^®^ Plus (OP), Bio-gide^®^ (BG), Jason^®^ membrane (JM), and sham operation (SO).

Membrane/Time Point	Day 10	Day 30	Day 60
CD163 (cells/mm^2^)			
OP	424.9 ± 17.8	507.5 ± 36.5	490.6 ± 65.7
BG	285.7 ± 67.9	686.5 ± 273.9	267.7 ± 123.5
JM	571.7 ± 145.3	219.4 ± 125.8	179.9 ± 144.3
SO	550.6 ± 76.1	824.3 ± 56.0	792.7 ± 116.3
CD11c (cells/mm^2^)			
OP	122.9 ± 122.3	52,9 ± 29.0	53.0 ± 2.0
BG	29.3 ± 14	23.8 ± 21.2	58.2 ± 29.1
JM	14.1 ± 3.8	23.5 ± 1.9	50.0 ± 5.0
SO	40.4 ± 6.0	53.7 ± 27.3	56.4 ± 10.0

**Table 2:** Interindividual statistical differences between the M1 and M2 cell numbers within the implantation beds of the analyzed barrier membranes (Ossix^®^ Plus (OP), Bio-gide^®^ (BG), Jason^®^ membrane (JM) and the sham operation (SO) (* *p* ≤ 0.05, ** *p* ≤ 0.01, **** *p* ≤ 0.0001, ns: not significant).

Time Points	Significance M1-M2
BG	
10D	**
30D	****
60D	*
OP	
10D	**
30D	****
60D	****
JM	
10D	****
30D	*
60D	ns
SO	
10D	****
30D	****
60D	****

**Table 3:** Concluding comparison of the investigated barrier membrane, Ossix^®^ Plus, and the controls, Bio-Gide^®^ and Jason^®^ membrane.

Membranes/Characteristics	OP [28]	BG [30]	JM [31]
Origin	Porcine (collagen type I)	Porcine (collagen type I and III)	Porcine (collagen type I and III)
Tissue Origin	Tendon	Skin	Pericardium
Production	Repolymerization	Decellularized tissue	Decellularized tissue
Crosslinking	Sugar crosslinked	No	No
Structural remarks	Nonporous	Bilayer (a porous layer and a compact layer)	Honeycomb-like porosity
Thickness	182 ± 13.6 µm	371 ± 31.4 µm	183 ± 6.9 µm
Initial swelling In Vivo	241.3%	254.1%	96.5%
Integration Pattern	No cellular infiltration	Cellular infiltration	Cellular infiltration
Transmembraneous Vascularization	No	Yes	Yes, slight
Occurrence of BMGCs	No	Yes (within the porous layer)	No

## Data Availability

All data presented in this study are integrated within the manuscript.
